# Mineral Trioxide Aggregate (MTA) Upregulates the Expression of *DMP1* in Direct Pulp Capping in the Rat Molar

**DOI:** 10.3390/ma14164640

**Published:** 2021-08-18

**Authors:** Maiko Yamada, Motohiko Nagayama, Yuka Miyamoto, Satoshi Kawano, Yoshiaki Takitani, Masashi Tanaka, Michiko Ehara, Juna Nakao, Takanaga Ochiai, Yoshihiro Shibukawa, Takakazu Yoshida

**Affiliations:** 1 Department of Endodontics, Division of Oral Functional Science and Rehabilitation, School of Dentistry, Asahi University, Mizuho, Gifu 501-0296, Japan; me_llamo_mai_00120_205@yahoo.co.jp (M.Y.); s-kawano@dent.asahi-u.ac.jp (S.K.); takisan@dent.asahi-u.ac.jp (Y.T.); m-tanaka@dent.asahi-u.ac.jp (M.T.); takakazu@dent.asahi-u.ac.jp (T.Y.); 2 Department of Oral Pathology, Division of Oral Pathogenesis and Disease Control, School of Dentistry, Asahi University, Mizuho, Gifu 501-0296, Japan; miyamoto@dent.asahi-u.ac.jp (Y.M.); ehara@dent.asahi-u.ac.jp (M.E.); nakao@dent.asahi-u.ac.jp (J.N.); t-ochiai@dent.asahi-u.ac.jp (T.O.); 3 Department of Removable Partial Prosthodontics, Tokyo Dental College, Chiyoda-Ku, Tokyo 101-0061, Japan; qh5y-sbkw@asahi-net.or.jp

**Keywords:** mineral trioxide aggregate, direct pulp capping, mineral density, nestin, dentin matrix acidic phosphoprotein 1 (*DMP1*), dentin bridge

## Abstract

Mineral trioxide aggregate (MTA) is an alternative endodontic material that predicts conductive or inductive calcified tissue formation from immature pulp mesenchymal stem cells (IPMSCs). The purpose of this study was to investigate whether MTA could promote reparative odontoblast differentiation via IPMSCs in the early phase of regeneration and compare with calcium hydroxide (CH). Direct pulp capping using calcium hydroxide (CH), MTA, and MTA with platelet-rich plasma (MTA + PRP) was performed on maxillary first molars of 8-week-old male Wistar rats (*n* = 36). After 3, 7, or 14 days, the teeth were analyzed for mineral density (MD) and volume of MD (VMD) via micro-focusing computed tomography (µCT), nestin, dentin matrix acidic phosphoprotein 1 (*DMP1*) immunohistochemistry, and real-time PCR for *DMP1* mRNA expression. MTA stimulated the early phase differentiation of the IPMSCs into odontoblasts, with positive results for nestin and *DMP1* compared with CH. Moreover, MTA + PRP stimulated calcified granule and dentin bridge formation through calcium mineral deposition, following the induction of *DMP1* mRNA expression in IPMSCs. Our results suggested that the combination of MTA and PRP is an effective and clinically applicable method for activating endogenous dental pulp stem cells into odontoblasts in the early stages of pulp regeneration.

## 1. Introduction

Direct pulp capping maintains the vitality and function of the dental pulp following its exposure to the external environment. Calcium hydroxide-based materials have been extensively used for this therapy because of their potential to induce hard-tissue repair and subsequent dentin bridge formation [1]. Recently, mineral trioxide aggregate (MTA) has received much attention as a good substitute for calcium hydroxide-based materials, and has demonstrated promising clinical outcomes [2]. MTA research has been focused on the biocompatibility and physical properties suitable for dental application, such as its excellent sealing ability [3].

MTA is a bioactive material developed in the early 1990s, initially a retrograde filling material, that first appeared in the dental scientific literature in 1993 [3]. Recently, MTA has been used to seal exposed pulp tissue and the root canal system from surrounding tissues for various applications, such as root-end filling, perforation repair, and apexification [4,5]. MTA comprises a modified preparation of 70 wt % of Portland cement, the basic ingredient of concrete and mortar; 20 wt % of bismuth oxides for radiopacity; and 5 wt % of gypsum [6,7,8,9]. However, we do not yet fully understand how to induce hard-tissue repair of the exposed pulps.

Dentin matrix acidic phosphoprotein 1 (*DMP1*) is one of the dentin non-collagenous extracellular matrix proteins implicated in bone mineralization regulation. This protein has the potential role of inducing cytodifferentiation of immature pulp mesenchymal stem cells (IPMSCs) into odontoblasts and the formation of reparative dentin in the rat model [10]. *DMP1* is also accepted as a newly formed dentin marker for odontoblasts and odontoblast-like cells [11,12,13]. It has been reported that *DMP1* and nestin can induce the differentiation of IPMSCs into odontoblasts [14,15,16]. However, this diversity and the role in the induction of the differentiation following reparative dentin or dentin bridge formation remain unclear.

This study aims to examine the biological process of pulp tissue reparative dentinogenesis by MTA.

## 2. Materials and Methods

### 2.1. Experimental Animals and Ethics

Thirty-six 8-week-old Wistar male rats (Chubu Kagaku Shizai Co., Ltd., Nagoya, Japan) were used in this study. The experimental animal protocol obtained approval according to the guidelines for animal care and ethics of Asahi University (10-013). 

### 2.2. Rat Molars and Direct Pulp Capping

The experiment was carried out according to the literature [17,18]. Rats were anesthetized by intraperitoneal injection of 5% pentobarbital sodium (Somnopentyl; Kyoritsu Seiyaku Co, Tokyo, Japan) and placed on the operating table with a manufactured fine rubber-dam condition to avoid bacterial infection. The dental pulp on the occlusal fissure area of bilateral maxillary first molars was exposed by diamond bur and then irrigated with a solution of 3% sodium hypochlorite, 3% hydrogen peroxide, and saline. After hemorrhage was controlled with sterile paper points, pulp capping was performed on exposed pulp using the following materials: calcium hydroxide (CH, Dycal^®^, Dentsply, York, PA, USA), mixed with the same amount of catalyst and base paste on the provided paper, 0.5 g powder form white-tooth-colored MTA (MTA, ProRoot MTA^®^, Dentsply, York, PA, USA) mixed with 0.2 mL of saline on the glass plate for one minute, or 0.5 g powder of MTA and 0.2 mL of platelet-rich plasma (MTA + PRP). PRP was extracted from allogeneic rat serum with the double-spin method (939× *g*, 10 min and 2113× *g*, 15 min) under 3.8 wt % sodium citrate condition. The mixed materials were placed on the exposed pulp surface with the metallic applicator. The pulp was then covered with adhesive resin (Multibond, Tokuyama Dental, Tokyo, Japan) and left for one hour (1 h) as control, 3 days, 7 days, and 14 days after pulp capping, respectively. For real-time reverse transcriptase PCR (RT-PCR) analysis, at 1 h and 3 days after direct pulp capping, the teeth were removed under anesthesia and total RNA was extracted by homogenizing at 4 °C (Polytron^®^, Kinematica, Bohemia, NY, USA) with 1 mL of extraction reagent (ISOGENⅡ, Nippon Gene, Tokyo, Japan). For histological and immunohistochemical studies, at 3 days, 7 days, and 14 days after direct pulp capping, rats were sacrificed by transcardial vital perfusion with 4% paraformaldehyde under anesthesia, and the maxilla, including the first molars, were dissected and immersed in 4% PFA at 4 °C for 12 h for further fixation.

### 2.3. Mineral Density Images and Analysis Using Micro-Focusing CT

At 7 days and 14 days after pulp capping, the first maxillary molars were examined by micro-focusing computed tomography (µCT, Comscan, Yokohama, Japan) under the following conditions: 90 V, 89 μA, 10-fold magnification, binning mode: 1, projection: 900. Photographic mineral density (MD) images were created with TRI Bon3D software (Ratoc System Engineering, Tokyo, Japan). MD data were examined as MD volume (VMD) with reference to the standard phantom material (Ratoc System Engineering, Tokyo, Japan).

### 2.4. Histological and Immunohistochemical Studies

Dissected maxillary bones were demineralized in 10% ethylenediaminetetraacetic acid (EDTA) in Tris buffer, pH 7.3 for 2 weeks with shaking. Demineralized specimens were dehydrated by upgrading a series of ethanol and xylene and embedded in paraffin, then sliced into 4 μm thick sections for hematoxylin–eosin staining (H–E) or immunohistochemistry. Immunohistochemistry was performed using anti-nestin mouse monoclonal antibody (CST, Danvers, Essex, MA, USA) and anti-*DMP1* rabbit polyclonal antibody (Takara Bio Inc., Shiga, Japan) at a dilution of 2 μg/mL each, and horseradish peroxidase polymer secondary antibodies (Histofine Simple Stain Rat MAX PO for rabbit and mouse IgG, Nichirei, Tokyo, Japan). The chromogenic activity of the peroxidase substrate was examined using diaminobenzidine (DAB) with hydroxide as an agitation.

### 2.5. Real-Time RT-PCR for DMP1 mRNA Expression

Relative quantitative analysis of *DMP1* mRNA expression at 3 d after pulp capping compared with the 1 h control was performed by real-time RT-PCR. The maxillary first molars were extracted under anesthesia and immediately subjected to total RNA extraction using a homogenizer (POLYTRON, Model K, PT10-35, KINEMATICA, Lucerne, Switzerland) with 1 mL of phenol and guanidine thiocyanate (ISOGEN, Nippon Gene, Tokyo, Japan). After dissolving, total RNA was proceeded to reverse transcription into cDNA using a commercial kit (High-Capacity cDNA Reverse Transcription Kit, Life Technologies, Carlsbad, CA, USA). The cDNA was examined by real-time PCR using primers for target rat *DMP1* (Rn01450122_mL); the housekeeping normalizer used was β-actin (Rn00667869_mL) (TaqMan^®^ probe and StepOne™ Real-Time PCR System, Life Technologies, Carlsbad, CA, USA). The comparative Ct (∆∆Ct) were calculated by threshold cycle (Ct), ∆Ct (sample target Ct − normalizer Ct), and shown as relative quantity (RQ) against the 3 days normal teeth without intervention (negative control).

### 2.6. Statistical Analysis

Statistical analysis was performed with Prism 9 (Prism 9 for macOS, GraphPad Software, San Diego, CA, USA). The results were subjected to a Mann–Whitney *U* test with the statistical null hypothesis that there was no difference between the experimental control group and each experimental group or between experimental groups. The significant comparison between every two groups was tested at a risk rate of *p* < 0.05.

## 3. Results

### 3.1. Photographic MD Images and VMD Differences

**Figure 1:**
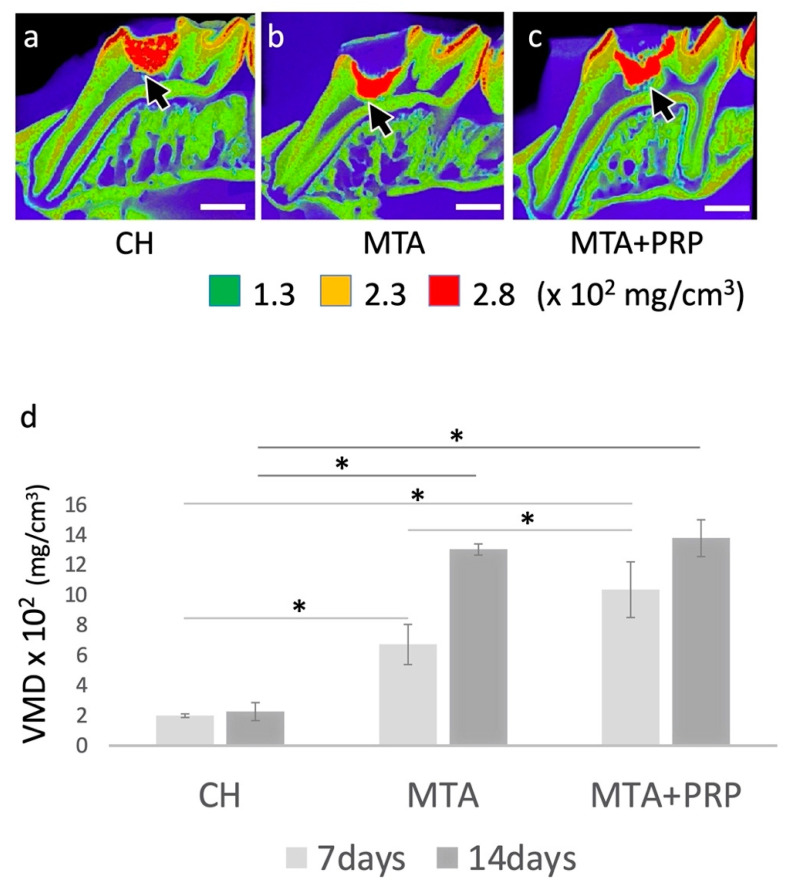
The µCT images with mineral density (MD) at 14 days, and MD volume (VMD) at 7 days and 14 days after pulp capping. High MD images (arrows) were found under the pulp-capping materials at 14 days after direct pulp capping: (**a**) CH; (**b**) MTA; (**c**) MTA + PRP. Note that both MTA and MTA + PRP were filled uniformly; however, CH was filled heterogeneously. (**d**) Each property of the dentin bridges beneath the pulp-capping materials showed various VMD values. Scale bars: 1.0 mm; * *p* < 0.05.

The µCT photograph with MD colored images showed slight differences between capping materials at 7 days (data not shown). However, at 14 days after direct pulp capping, dentin bridge formation was significantly different, especially regarding the thickness. VMD values from the dentin bridge area of CH, MTA, and MTA + PRP treatments were 199 ± 9.85 (mg/cm^3^), 673 ± 133.68 (mg/cm^3^), and 1037 ± 185.00 (mg/cm^3^) at 7 days, and 227 ± 49.09 (mg/cm^3^), 1304 ± 37.35 (mg/cm^3^) and 1380 ± 122.50 (mg/cm^3^) at 14 days, respectively. Quantitative analysis revealed that the VMD of the dentin bridges formed by CH treatment was statistically lower than that of MTA and MTA+PRP treatment at both 7 days and 14 days. On the other hand, a statistical difference between the MTA and MTA+PRP treatments was only observed at 7 days (Figure 1).

### 3.2. Histological and Immunohistological Findings

**Figure 2:**
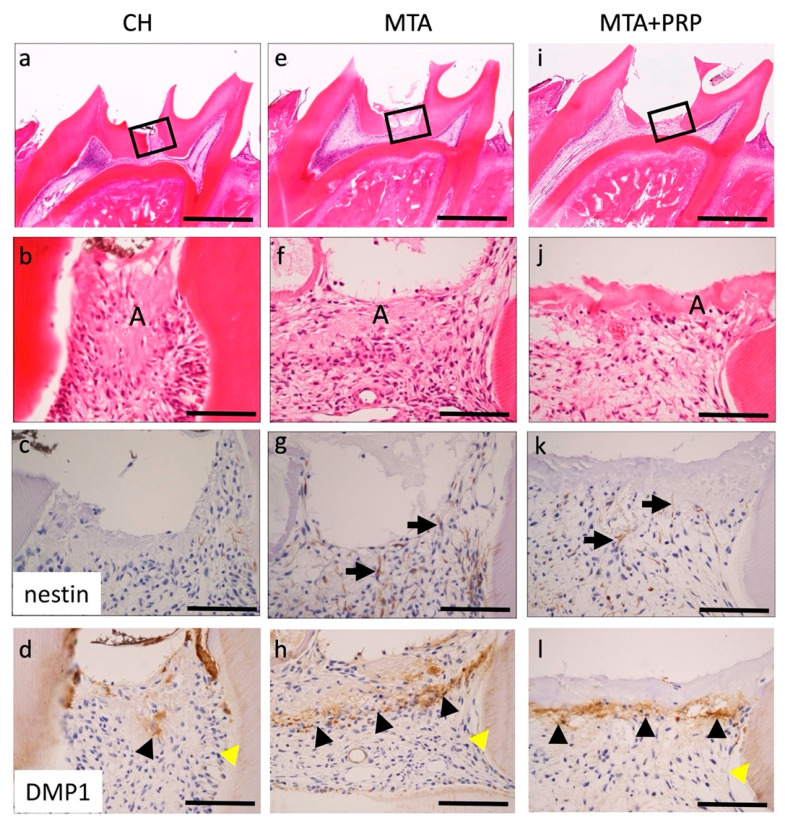
Histological findings of the pulp tissue at 3 days after pulp capping with CH (**a**–**d**), MTA (**e**–**h**), or MTA + PRP (**i**–**l**). Necrotic or amorphous layers (**A**) were observed at the exposure site of the dental pulp with relative inflammatory cell infiltration (**b**,**f**,**j**). Nestin was negative at the exposure site of CH (**c**). However, fibrillar and spindle-shaped cells were positive at the exposure site of MTA and MTA + PRP (**g**,**k**, black arrows). *DMP1* was localized in the dentinal tubes (**d**,**h**,**l**, yellow arrowheads) and extracellular matrix beneath the necrotic/amorphous layers at the exposure site of CH, MTA, and MTA + PRP (**d**,**h**,**l**, black arrowheads). Scale bars: 1 mm in (**a**,**e**,**i**); 100 μm in (**b**–**d**,**f**–**h**,**j**–**l**).

Histological changes in the pulp tissue at 3 days, 7 days, and 14 days after pulp capping with CH, MTA, and MTA + PRP were shown in the H–E and immunohistochemistry for nestin and *DMP1*. At 3 days, thin necrotic or amorphous layers were observed beneath the CH, MTA, and MTA + PRP, and relative edematous and inflammatory cell migrations or exudative phases were observed. Immunohistochemically, nestin was not observed at the exposure of the CH site. However, odontoblasts in other parts of the pulp tissue were positive for nestin. Several fibrillar and spindle-shaped cells that were nestin-positive were localized beneath the necrotic or amorphous layers of MTA and MTA + PRP. *DMP1* was localized in the extracellular matrix beneath the necrotic or amorphous layers. These positive intensities were enhanced in the order of CH < MTA < MTA + PRP (Figure 2; Table 1).

**Figure 3:**
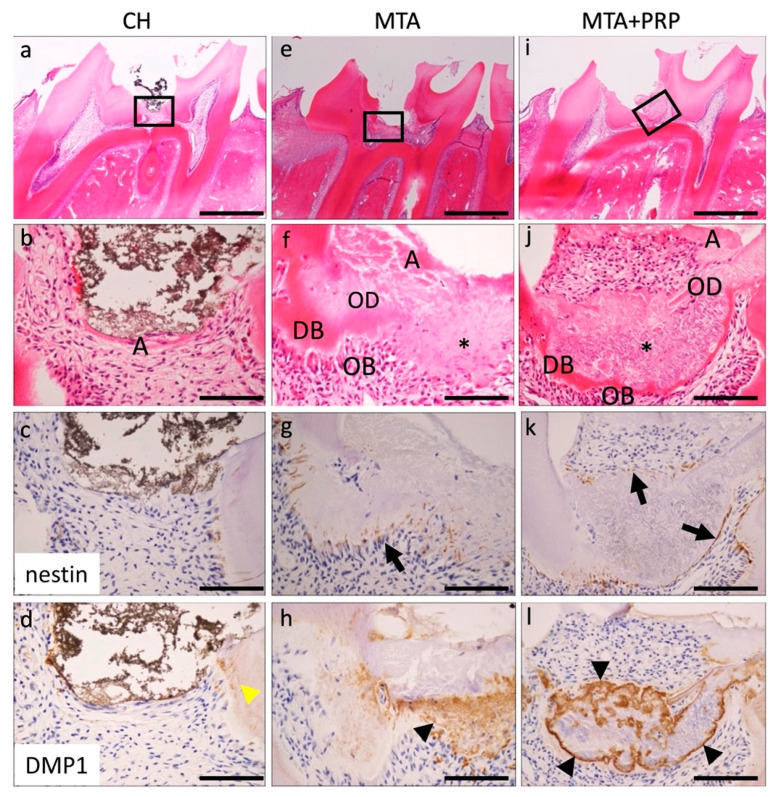
Histological findings of the pulp tissue at 7 days after pulp capping with CH (**a**–**d**), MTA (**e**–**h**), or MTA + PRP (**i**–**l**). Necrotic or amorphous layers (**A**) were observed at the exposed side of the dental pulp with few inflammatory infiltrations (**b**,**f**,**j**). In the deep layer of pulp capping with MTA and MTA + PRP, the fine granular extracellular matrix (*) and eosinophilic DB or OD with OB lining were observed. Nestin was negative in CH (**c**); however, apparent nestin-positive OBs were lined under the DB in MTA and MTA + PRP (**g**,**k**, black arrows). *DMP1* was only localized in the dentinal tubes of the primary dentin in CH (**d**, yellow arrowhead). However, a prominent *DMP1*-positive matrix was observed along with the outer layer of the DB in the MTA and MTA + PRP (**h**,**l**, black arrowheads). DB, dentin bridge; OD, osteoid dentin; OB, odontoblast; Scale bars: 1 mm in (**a**,**e**,**i**); 100 μm in (**b**–**d**,**f**–**h**,**j**–**l**).

At 7 days, a secreting tissue regeneration phase was observed in the area treated with MTA and MTA + PRP. The necrotic or amorphous layers were still observed beneath the MTA and MTA + PRP. Interestingly, the fine granular extracellular matrix and exhibited eosinophilic dentin bridge (DB) or osteoid dentin (OD) formation, which included cells in the formed matrix and lining odontoblasts, were observed beneath the necrotic or amorphous layers with few inflammatory infiltrations. As a characteristic change, the fine-grained extracellular matrix was continuously observed in the eosinophilic dentin bridge (DB) or osteoid dentin (OD) lined with odontoblasts, but no dentinal tube-like structure was observed. Nestin was not observed at the exposure of the CH site, except in the odontoblasts of the normal predentin. However, several odontoblasts beneath the fine granular extracellular matrix and dentin bridge treated with MTA and MTA + PRP were nestin-positive. *DMP1* was localized in the dentinal tubes of the primary dentin in CH and around the fine granular extracellular matrix of DB or OD (Figure 3; Table 1).

**Figure 4:**
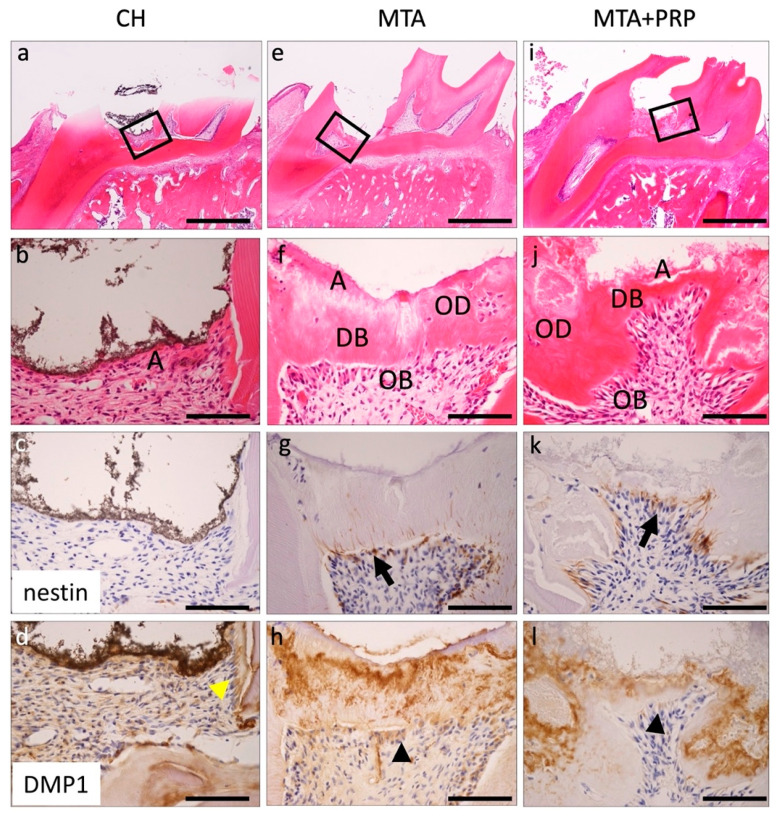
Histological findings of the pulp tissue at 14 days after pulp capping with CH (**a**–**d**), MTA (**e**–**h**), or MTA + PRP (**i**–**l**). Necrotic or amorphous layers (**A**) were observed at the exposure site of the dental pulp without inflammatory cell infiltration (**b**,**f**,**j**). The fine granule extracellular matrix was no longer seen in the deep layer of pulp capping with MTA and MTA + PRP. However, the eosinophilic reparative DB or OD with OB lining was observed. Nestin was negative in the CH (**c**). However, apparent nestin-positive OBs were lined under the DB or OD in MTA and MTA + PRP (**g**,**k**, black arrows). *DMP1* was localized at the odontoblasts of the primary dentin in CH (**d**, yellow arrowhead). However, a positive matrix area was spread into DB or OD and osteoblasts in the MTA and MTA + PRP (**h**,**l**, black arrowheads). DB, dentin bridge; OD, osteoid dentin; OB, odontoblast; Scale bars: 1 mm in (**a**,**e**,**i**); 100 μm in (**b**–**d**,**f**–**h**,**j**–**l)**.

At 14 days, this reparative hard tissue formation—necrotic or amorphous layers (Figure 3a)—was still observed at the exposure site of the dental pulp, but inflammatory infiltration was no longer seen. The eosinophilic reparative DB or OD and odontoblasts lining were observed in the deep layer of pulp capping with MTA and MTA + PRP. The fine-grained extracellular matrix seen at 7 days was no longer observed. However, delicate dentin tube-like structures of eosinophilic dentin bridges (DB) and osteoid dentin (OD) lined with odontoblasts were observed. Nestin was negative in the CH; however, apparent nestin-positive spindle-shaped odontoblasts were lined under the DB or OD in MTA and MTA + PRP. *DMP1* was prominent positive at the DB or OD in MTA and MTA + PRP (Figure 4; Table 1). 

### 3.3. DMP1 mRNA Expression

**Figure 5:**
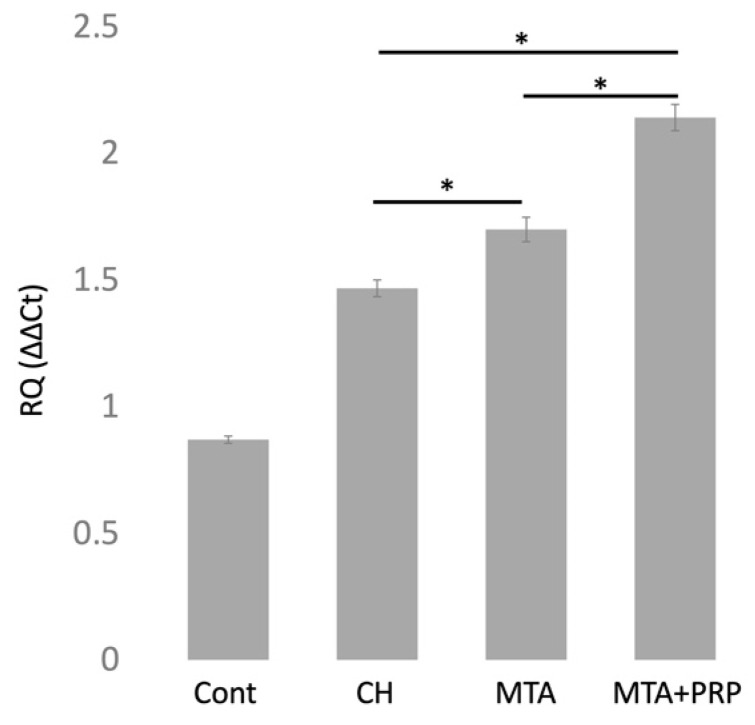
Relative quantitative (RQ) value of *DMP1* mRNA expression at 1 h (control) and 3 days (CH, MTA, MTA + PRP) after pulp capping. In the RQ (∆∆CT) condition, *DMP1* mRNA was expressed in the order of CH < MTA < MTA + PRP, which was similar to the findings of *DMP1* immunohistochemistry. * *p* < 0.05.

Relative quantitative measurement of *DMP1* mRNA expression using the ∆∆CT method at 3 days after pulp capping with CH, MTA, and MTA + PRP compared with 1 h control. *DMP1* mRNA was expressed at higher levels in the order of CH < MTA < MTA + PRP (Figure 5).

## 4. Discussion

Dental pulp tissue possesses the potential for natural tissue repair, leading to reparative dentin formation. It has been well documented that dental pulp tissue forms a hard tissue, known as the dentin bridge, after direct pulp capping. During reparative dentinogenesis, the original odontoblasts and pulpal cells at the exposure site undergo necrosis and are gradually replaced by newly differentiated odontoblast-like cells [1,19,20,21]. In this study, we examined the effects of MTA on the acceleration of the early phases of dental pulp wound healing and their subsequent proliferation and differentiation into odontoblast-like cells to form the reparative dentin bridge. We also looked at the involvement of tissue already in the injured site.

It is well known that stem or progenitor cells, appropriate scaffolds, and growth factors are required to achieve effective tissue regeneration. In this study, we used residual amputated pulp tissue to induce IPMSCs, MTA as a scaffold, and PRP as the growth factor compared with CH. During reparative dentinogenesis, the adhesion of stem or progenitor cells to an appropriate scaffold surface may be critical for hard-tissue-forming cells [22]. When bioactive materials, including CH or MTA, are applied to the exposed pulp tissue, a layer of dystrophic calcification associated with cellular degeneration may be present on the surface, which causes the migration and attachment of IPMSCs to this site, and their subsequent differentiation into odontoblast-like cells.

The formation of dentin-bridge-like hard tissue was observed in the MD images and VMD values after 14 days with MTA and MTA+PRP filling, but the formation and VMD values were low in CH. The cavities in the CH material were also observed, suggesting that the filling of CH was uneven and poorly sealed [6,7].

Histologically, after 3 days of filling with MTA and MTA+PRP, DB and OD formation were not observed, while nestin-positive fibrous spindle-shaped odontoblasts were found directly under the material. After 7 days and 14 days, the fine-grained extracellular matrix was observed and positive for *DMP1* in the DB or OD. These *DMP1*-positive granular matrices were eventually deposited in the dentin tubes of DB and OD. It was suggested that the *DMP1* positive matrix, which was lacking at 3 days, may be associated with hard tissue formation in DB and OD, but further investigation is needed.

Nestin is an intermediate filament protein that has been proposed as a marker for differentiated odontoblasts [15,16]. In tooth buds of humans and rodents, nestin is widely distributed not only in odontoblasts, but also in the dental pulp, especially in the cusp [15,16]. In mature teeth, nestin is present only in functioning odontoblasts, and nestin expression decreases during tooth maturation [15,16]. However, nestin is reactivated and its expression increases in the dentin/pulp complex under pathological conditions caused by the preparation of dental caries [16]. Because nestin expression is a temporospatial variable, this study used immunohistochemistry for nestin to detect differentiated odontoblasts instead of real-time RT-PCR. Nestin-positive differentiated odontoblasts were observed at the site of pulp amputation beneath the necrotic or amorphous layer following direct capping with MTA and MTA + PRP. This nestin localization revealed that re-expressed activity of nestin depends on the pulp-capping materials. According to our data, tissue regeneration or repair reaction is divided into three stages: exudate or inflammation without bacterial infection, and the cellular migration and differentiation phase appearing at around 3 days; the extracellular matrix secretion phase at around 7 days; and the hard-tissue formation of maturation phase at around 14 days. These findings suggested that MTA and MTA + PRP may have inducible odontoblast differentiation of IPMSCs to odontoblasts via some growth factors, such as platelet-derived growth factor (PDGF) in PRP. However, CH may be low or take more time to form.

Cellular calcification, such as odontogenesis or reparative dentin formation, requires mineral deposition on the matrix after matrix vesicle secretion. More than 90% of the extracellular matrix of dentin is collagen type I fibers, which provide the structural framework that defines compartments for ordered mineral deposition [23]. However, it is well established that collagen alone cannot induce matrix-specific mineral formation from metastable calcium phosphate solutions that do not spontaneously precipitate [24]. Thus, the non-collagenous matrix components that compound the remaining 10% of the organic dentin matrix are required to initiate calcification. The non-collagenous matrix components comprise a number of highly phosphorylated proteins that possess calcium-binding properties and function either as promoters or as inhibitors of mineral deposition in in vitro experiments [25]. Such proteins include the following: dentin sialophosphoprotein (DSPP), a large parental protein that subsequently suffers cleavage to form two products, dentin sialoprotein (DSP) and dentin phosphoprotein (DPP); dentin matrix protein (DMP) 1, DMP2, and DMP3; osteopontin (OPN); bone sialoprotein (BSP); osteonectin; and osteocalcin [26,27,28]. These non-collagenous proteins may be associated with the initially formed calcified layer underneath the superficial necrotic layer. *DMP1* is a major acidic non-collagenous matrix protein first cloned from the mineralized dentin matrix [29] and later from the bone matrix [30].

Current studies focus on the derivation and phenotypes of the cells involved in nonspecific reparative dentinogenesis and the molecular mechanisms that regulate their cell differentiation. Our findings indicated that the MTA-induced reparative dentinogenesis process is similar to the process of CH, which follows the proliferation, migration, and differentiation of progenitor cells before matrix secretion at the exposure site [1]. This might be attributed, at least in part, to the fact that CH is formed in MTA during setting [31,32]. Thus, the primary process of reparative dentinogenesis after MTA capping might involve the natural pulpal wound-healing mechanism [33]. However, MTA caused mild inflammatory and necrotic changes in the sub-adjacent pulp at 3 days after pulp capping. This is contrary to CH, which is known to cause extensive pulp tissue necrosis and inflammatory cell infiltration shortly after its application [1]. Thus, MTA might be less caustic than traditional CH preparations. Such approaches will ultimately lead to regenerative therapy and tissue engineering of the dentin–pulp complex.

Moreover, a recent in vitro study established that MTA and PRP can initiate the pulp tissue’s regeneration activity via IPMSCs [34]. Our results showed a similar tendency to those of previous studies on direct pulp capping, but the combination of MTA and PRP seemed to promote the differentiation of IPMSCs into odontoblasts and dentin bridge hard-tissue formation in the early stage of treatment [35]. Various novel materials, including MTA, for direct pulp capping have been summarized [36]. The development of dental pulp regenerative therapy requires improving materials and unique ideas for the clinical approach. For example, pre-mixing materials and growth factors such as MTA and PRP are expected to be developed in future clinical approaches.

## 5. Conclusions

This study demonstrated that MTA could induce hard-tissue formation at an early stage of pulp regenerative therapy. In addition, the expression of *DMP1* could be an indicator. This mechanism, however, remains unresolved. The development of pulp regeneration therapy requires not only the improvement of materials, but also unique clinical ideas.

## Figures and Tables

**Table 1:** Summary of the histological and immunohistochemical findings.

After Pulp Capping	3 Days			7 Days			14 Days		
Pulp Capping Materials	CH	MTA	MTA + PRP	CH	MTA	MTA + PRP	CH	MTA	MTA + PRP
DB/OD	0	0	0	0	1	1	0	2	2
nestin	0	1	1	0	1	1	0	2	2
*DMP1*	1	1	1	0	1	2	0	1	2

Note: Score 0, negative; score 1, relative positive intensity; score 2, apparent positive intensity; CH, calcium hydroxide; MTA, mineral trioxide aggregate; MTA + PRP, MTA with platelet-rich plasma; DB/OD, dentin bridge/osteoid dentin.

## Data Availability

The data presented in this study are available on request from the corresponding author. The data are not publicly available due to privacy.
